# Strategies to Overcome Antigen Heterogeneity in CAR-T Cell Therapy

**DOI:** 10.3390/cells14050320

**Published:** 2025-02-20

**Authors:** Bohan Zhang, Jiawen Wu, Hua Jiang, Min Zhou

**Affiliations:** 1 State Key Laboratory of Systems Medicine for Cancer, Shanghai Cancer Institute, Renji Hospital, Shanghai Jiaotong University School of Medicine, Shanghai 200032, China; bohan6309@sjtu.edu.cn (B.Z.); wjw-2001@sjtu.edu.cn (J.W.); 2 CARsgen Therapeutics, Shanghai 200231, China

**Keywords:** CAR-T, antigen heterogeneity, TME, endogenous immunity

## Abstract

Chimeric antigen receptor (CAR) gene-modified T-cell therapy has achieved significant success in the treatment of hematological malignancies. However, this therapy has not yet made breakthroughs in the treatment of solid tumors and still faces issues of resistance and relapse in hematological cancers. A major reason for these problems is the antigenic heterogeneity of tumor tissues. This review outlines the antigenic heterogeneity encountered in CAR-T cell therapy and the corresponding strategies to address it. These strategies include using combination therapy to increase the abundance of target antigens, optimizing the structure of CARs to enhance sensitivity to low-density antigens, developing multi-targeted CAR-T cells, and reprogramming the TME to activate endogenous immunity. These approaches offer new directions for overcoming tumor antigenic heterogeneity in CAR-T cell therapy.

## 1. Introduction

Chimeric antigen receptor T cell therapy (CAR-T therapy) is a cutting-edge cellular immunotherapy method. It involves introducing the chimeric antigen receptor (CAR) gene into the patient’s or donor’s T cells, enabling the T cells to overcome major histocompatibility complex (MHC) restriction and directly target tumor surface antigens, thereby exerting cytotoxic effects [1]. Currently, the CAR-T products approved for marketing are primarily used to treat hematological malignancies, and substantial breakthroughs in the field of solid tumors have yet to be made [2,3,4]. CAR-T therapy still faces numerous challenges, including T-cell dysfunction, immunosuppressive microenvironment, and antigen heterogeneity [5,6,7]. Particularly, the spatiotemporal heterogeneity and individual variability of tumor surface antigens remain major obstacles limiting the effectiveness of CAR-T therapy. This article discusses the evolutionary dynamics of both CAR-T cells and tumor cells during their interaction and summarizes the current strategies to address antigen heterogeneity. These strategies include using combination therapies to induce or maintain target antigen expression, optimizing CAR structure to improve the sensitivity of CAR-T cells to low-density antigens, designing multi-target CAR-T cells to improve the killing ability of CAR-T cells against heterogeneous tumors, introducing various immune-stimulatory elements into CAR-T cells, and combining CAR-T cells with oncolytic viruses, probiotics and vaccines to actively remodel the immunosuppressive tumor microenvironment (TME) and stimulate endogenous immune responses, thereby increasing the killing of antigen-negative cancer cells.

## 2. Antigen Heterogeneity in CAR-T Therapy

A prerequisite for successful CAR-T treatment is the correct recognition and interaction between the single-chain variable fragment (scFv) in CAR and tumor-specific antigens (TSA) or tumor-associated antigens (TAA) [8]. However, tumor antigens are highly heterogeneous, both in type and quantity [9,10]. This heterogeneity is particularly challenging in solid tumors, where finding a uniformly expressed antigen on all tumor cells is difficult [11]. As a result, CAR-T cells are usually unable to eliminate all tumor cells effectively, creating opportunities for relapse and treatment resistance, which contributes to inherent resistance in CAR-T therapy. Additionally, CAR-T cells and cancer cells mutually reshape each other during their interaction. Tumor cells undergo immune editing effects induced by CAR-T, dynamically remodeling their antigen expression profile [12,13,14,15]. This manifests as the selective expansion of antigen-negative tumor cells present before treatment and the re-editing of antigens on antigen-positive cells, leading to acquired resistance. For CAR-T cells, this interaction can result in CAR downregulation and increased expression of immunosuppressive molecules [16,17,18], forming a target-related escape mechanism.

### 2.1. The Phenomenon and Mechanism of Tumor Antigen Escape After CAR-T Therapy

Tumors exhibit significant intra- and inter-tumoral antigen heterogeneity, both in terms of antigen diversity and uneven distribution of the same antigen [9]. The efficacy of CAR-T cell killing relies on high antigen density, while low antigen density is associated with resistance and relapse after CAR-T treatment [19,20,21]. Additionally, antigen loss is one of the primary causes of CAR-T therapy resistance and relapse. In clinical trials targeting antigens such as CD19, CD22, BCMA, and GPRC5D for hematologic malignancies, a decrease in target antigen density has been observed in relapsed patients [12,19,22,23,24]. Similar antigen escape phenomena have been observed in solid tumor treatment. Among seven patients treated with EGFRvIII-CAR-T for GBM, five showed a reduction in antigen expression after CAR-T cell infusion [25]. Likewise, following GD2-CAR-T treatment for GBM, GD2 antigen loss was observed in the resected tumor tissue [26].

An increasing number of studies have revealed antigen adaptation mechanisms following CAR-T cell therapy for hematologic malignancies, including lineage switching [27], selective RNA splicing [28], and gene mutations or loss of heterozygosity, leading to the loss of functional epitopes and transmembrane regions [12,13,14,15]. Post-translational modifications of proteins can also affect the recognition efficiency of CAR-T cells. Aberrant glycosylation of tumor antigens can resist CAR-T cell activity by masking CAR recognition epitopes or disrupting immune synapse formation [29,30]. Intervening in the glycosylation modifications on the surface of tumor cells can mitigate tumor resistance [30].

Interestingly, antigen transfer on the surface of tumors also occurs. Hamieh and colleagues discovered that CAR-T cells, through scFv recognition and trogocytosis, acquired antigens from the surface of tumor cells, which were then transferred to the surface of CAR-T cells [31]. This procedure promoted fratricide among CAR-T cells and accelerated T cell exhaustion [31]. It has been reported that using low-affinity CARs or attaching the cytoplasmic tail of CTLA-4 to the C-terminus of CAR may reduce CAR-T cell trogocytosis [32,33]. Lu and colleagues found that targeting the activating transcription factor-3 (ATF3)-cholesterol 25-hydroxylase (CH25H) regulatory axis could also limit trogocytosis [34]. In addition, pancreatic cancer cells secrete small extracellular vesicles (EVs) carrying PD-L1 and MSLN, which not only reduce antigen density but also interfere with the proper targeting of endogenous T cells and MSLN-CAR-T cells [35]. Inhibiting the secretion of tumor-derived EVs can enhance the efficacy of CAR-T cells [35].

### 2.2. CAR Downregulation

In the interaction with tumor cells, apart from the antigen downregulation on the cancer cell surface caused by immune pressure, CAR molecules on CAR-T cells can also be downregulated through various mechanisms, which affects their effective recognition of tumor cells [16,17]. As mentioned earlier, CAR-T cells can remove tumor antigens via trogocytosis, and a recent study has shown that tumor cells can also strip CARs from CAR-T cells through trogocytosis [16]. This effect is related to antigen density, the sensitivity of CAR molecules, and the cholesterol metabolism of tumor cells [16].

In T cell immunity, after antigen activation, the TCR/CD3 complex undergoes ubiquitin-mediated degradation, reducing the number of TCRs and decreasing the frequency of antigen-induced signaling to avoid T cell overactivation [36]. CARs also exhibit antigen-dependent downregulation. However, excessive CAR downregulation creates opportunities for tumor escape [21]. Preventing CAR internalization is a potential strategy to improve the persistence of CAR-T cell therapy and prevent tumor escape. Researchers successfully inhibited CAR ubiquitination and lysosomal degradation by mutating lysine residues in the CAR intracellular domain to arginine, which was beneficial to the recycling of endogenous CARs back to the cell surface and prevented CAR downregulation [17]. Additionally, CAR-T cells lacking CTLA-4 can maintain surface CAR expression under chronic antigen stimulation [37].

## 3. Combination Therapy Increases the Abundance of Target Antigens

**Figure 1:**
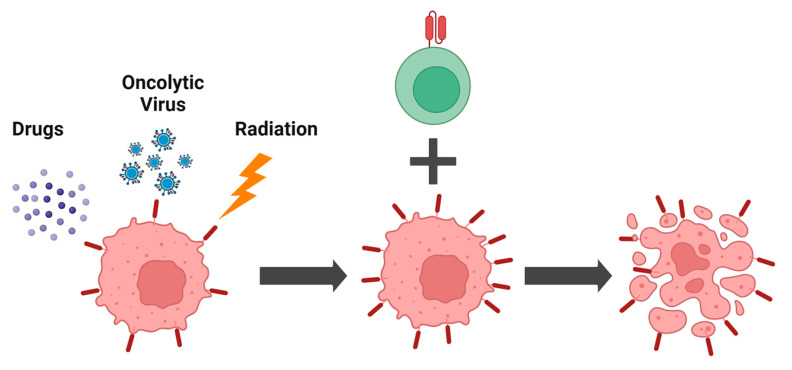
Combination therapy increases the abundance of target antigens, enabling more efficient killing of tumor cells. The cytotoxic efficiency of CAR-T depends on target antigen density. Drugs, oncolytic viruses, and radiation enhance the abundance of target antigens on the tumor cell surface.

Although tumor cell surface antigens may be downregulated due to immune editing, this process can be reversed through combination therapies, thereby increasing target antigen density (Figure 1). For example, reducing the degradation of target antigens is a common approach. BCMA, a target marker for multiple myeloma (MM), can be cleaved by γ-secretase (GS), reducing its surface density and generating soluble molecules that can bind to CAR receptors, thus limiting the efficacy of BCMA-targeted CAR-T cell therapy [38]. Therefore, using a γ-secretase inhibitor (GSI) can increase BCMA surface expression and enhance the efficacy of CAR-T cells [38]. A phase 1 clinical trial has demonstrated that the combination of GSI and BCMA CAR-T cells is well-tolerated and that GSI can increase target antigen density in vivo [39]. Roberto Chiarle’s team discovered that CAR-T cells targeting anaplastic lymphoma kinase (ALK) can effectively eliminate neuroblastomas with high ALK expression [40]. For neuroblastomas with low ALK expression, an ALK inhibitor can increase ALK expression on tumor cells by preventing ALK internalization and degradation, thereby enhancing CAR-T cell-mediated cytotoxicity against tumors [40].

Targeting signaling pathways is also a way to increase antigen expression. Lee et al. performed a high-throughput screening of 1114 FDA-approved drugs and found that ingenol-3-angelate (I3A) can increase B7-H3 surface abundance on osteosarcoma cells via the protein kinase Cα pathway [41]. Since CD72 is a negative regulator of the B-cell receptor (BCR) signaling pathway, activation of the BCR signaling pathway by specific drugs can promote CD72 expression in B-ALL cells through a negative feedback mechanism, thereby providing additional targets for CD72-directed CAR-T therapy [42].

Epigenetic control of target antigen expression is another strategy to flexibly regulate antigen density. Certain chemotherapeutic agents can modulate DNA demethylation, leading to the reactivation of key epigenetically silenced genes. For example, the demethylating agent 5-AZA can re-induce the expression of CD123 and CD70 on AML cells following treatment [43,44]. In addition, radiation therapy can induce the expression of tumor antigens such as mesothelin and enhance the anti-tumor effects of CAR-T cells [45,46].

Oncolytic viruses, which can selectively deliver transgenes to tumors, were leveraged by Park et al. to develop a virus expressing a truncated, non-signaling CD19 (CD19t) protein, which was then used to infect tumor cells [47]. Before virus-mediated tumor lysis, CD19t is expressed on the cell surface, addressing the issue of low antigen density. The lysed tumor cells subsequently facilitate the rapid release of internalized viral particles, thereby promoting the cascade-like dissemination of specific antigens within the tumor microenvironment [47].

In summary, understanding the dynamic expression processes of target antigens, targeting relevant nodes, or inducing antigen expression, and combining these strategies with CAR-T therapy are effective ways to address the challenge of low antigen density.

## 4. Optimizing the Structure of CARs to Enhance Sensitivity to Low-Density Antigens

Although the introduction of CAR has enabled CD8 T cells to overcome MHC restriction, the initial activation of CD8 T cells can be triggered by as few as 1-10 peptide-MHC complexes, while CAR activation requires hundreds to thousands of antigen molecules [48]. This difference in sensitivity is associated with a lower number of activated immunoreceptor tyrosine-based activation motifs (ITAMs) in CAR-T cells compared to TCRs following antigen stimulation, weaker downstream signals, such as CD3 ITAM, zeta chain of T cell receptor-associated protein kinase 70 (ZAP-70), and linker for activation of T cells (LAT) phosphorylation, as well as disorganized immune synapse structures [48,49]. Therefore, optimizing CAR structure to amplify CAR-T cell activation signals under low antigen density recognition conditions is a promising strategy for targeting tumors with low antigen expression.

The structure of a CAR primarily consists of a scFv that recognizes TSA or TAA, a transmembrane domain, intracellular costimulatory domains, and a CD3ζ signaling domain [1]. Typically, physicians select an appropriate target antigen based on its expression type and abundance in the patient. The scFv is the key component responsible for antigen recognition, comprising the variable light (VL) and variable heavy (VH) regions of an antibody connected by a linker, forming a single-chain structure with full antigen-binding capability. The first-generation CAR contained only a scFv for TSA or TAA recognition and a CD3ζ signaling domain. However, due to the absence of a costimulatory domain, it failed to effectively amplify signals upon antigen stimulation, limiting its cytotoxic function. To enhance signal transduction and T cell activation, second-generation CAR-T cells introduced a costimulatory domain (such as CD28 or 4-1BB). The third generation incorporated two or more costimulatory domains for further enhancement. To overcome challenges such as tumor antigen heterogeneity, an immunosuppressive microenvironment, infiltration difficulties, and T cell exhaustion, advanced CAR-T designs have incorporated additional regulatory gene modules to improve efficacy and safety [8,50]. The CAR gene is then integrated into autologous or allogeneic T cells using lentiviral, retroviral, or non-viral methods. Before the first human clinical trial, the modified CAR-T cells must undergo preclinical validation to ensure their safety and efficacy [51].

It has been reported that increasing the number of ITAMs can enhance the efficacy of CAR-T cells against tumor cells with low antigen density [52]. In tumor antigen escape models, CD28-based CARs demonstrated better control of CD19-low ALL relapse compared to 4-1BB-based CARs [52]. CARs containing CD28 hinge and transmembrane (H/T) domains exhibited greater sensitivity against tumors in low-antigen models [52,53], likely due to stronger receptor clustering and proximal signaling molecule recruitment, such as ZAP-70, in CD28 H/T-containing CARs [52]. Recent studies have also shown that the target antigen epitope recognized by the scFv can influence the antigen activation threshold and cytotoxicity [54,55]. In CAR-T cells targeting CD33 for the treatment of AML, scFvs targeting membrane-proximal epitopes of CD33 generated more robust cytotoxicity against low antigen abundance tumors compared to scFvs targeting membrane-distal epitopes [54].

**Figure 2:**
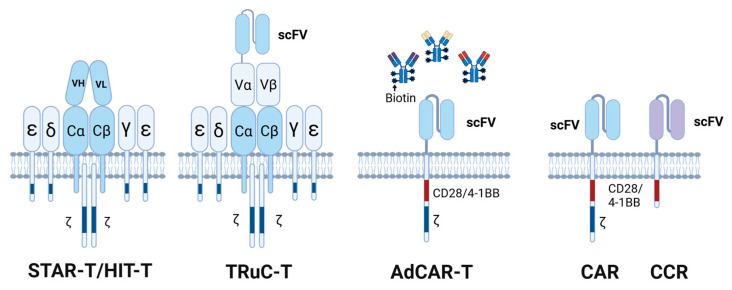
Modify CAR structure to enhance the killing of target cells with different antigen expression patterns. STAR, HIT, and TRuC couple the antigen recognition domain with the TCR structure. This design overcomes HLA restriction while retaining intact TCR signaling, ensuring more controlled signal transmission, lowering the antigen recognition threshold, and enhancing sensitivity to low-density antigens. In the AdCAR-T platform, CAR recognizes adapter molecules (e.g., biotin-labeled antibodies), while the variable regions of antibodies target different tumor antigens. This allows for flexible combinations targeting different antigen profiles. The CCR platform is characterized by CCR and CAR targeting different antigens, amplifying the CAR signal, and improving safety.

In addition, compared to the TCR complex, the signaling components of CAR are relatively simple (containing only one CD3ζ) [56]. Increasing research efforts are focusing on designing chimeric receptors that resemble the TCR structure or adding more TCR-related elements to achieve more efficient and balanced activation. Based on this concept, Liu et al. designed a novel synthetic T cell antigen receptor (STAR) T cell (Figure 2), which fuses the variable regions of the antibody’s light and heavy chains with the constant regions of the TCR’s α and β chains, thereby forming a complex with the endogenous CD3 subunits of T cells [57]. This conformation endows STAR-T cells with the high specificity and affinity of CARs, along with downstream signaling amplification properties of TCRs. In multiple solid tumor models, STAR-T cells demonstrated superior performance compared to BBzCAR-T cells and showed better or comparable anti-tumor effects compared to 28zCAR-T cells, effectively addressing the issue of low antigen expression [57]. Similarly, HIT (HLA-independent T), established by the integration of scFv into the TCR/CD3 complex (Figure 2), can also recognize and kill tumor cells with low target antigen expression independent of HLA [58]. Additionally, the TCR fusion construct T cell (TRuC-T, Figure 2) was developed by directly linking scFv to the CD3ε subunit of TCRs [59]. Overall, chimeric receptors with more sophisticated signaling modules have demonstrated higher stability and efficacy in cytotoxicity control.

Traditional CARs are designed to target tumor-specific antigens, making them vulnerable to spatiotemporal antigen variability. Adapter CAR-T (AdCAR-T) offers a novel approach to address this challenge. In this platform, one end of the adapter is labeled with biotin that can be recognized by the CAR, and the other end targets various AML surface antigens (Figure 2). The AdCAR-T system allows for the adjustment of adapter molecule quantity and combinations as needed, enabling precise targeting of different individuals and providing higher flexibility and controllability compared to single-targeted CAR. It was shown that AdCAR-T cells could effectively recognize and eliminate AML cells expressing different antigens, thereby avoiding immune evasion caused by antigen heterogeneity [9,60].

One of the reasons that CAR-T cells are ineffective in killing tumor cells with low antigen density is due to insufficient activation signals, which fail to fully activate downstream signaling pathways. Additionally, amplifying a single antigen signal may lead to off-target toxicity [1]. The design of chimeric co-stimulatory receptors (CCR) addresses both of these issues. CCR and CAR target different antigens (Figure 2), and their coordinated recognition amplifies the antigen stimulation signal, promoting full T cell activation [61,62]. The inclusion of CCR makes CARs more sensitive and flexible, allowing them to adapt to the heterogeneous spectrum of antigen expression.

## 5. Multi-Targeted CAR-T Therapy

**Figure 3:**
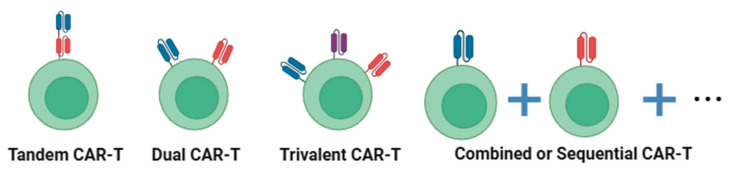
Enhance tumor surface antigen coverage through multi-targeted therapy. Tandem CAR-T expresses scFv targeting different antigen epitopes in the same CAR molecule. Dual-target CAR-T and trivalent CAR-T independently express different CAR molecules in CAR-T cells. Simultaneous or sequential infusion of multiple single-targeted CAR-T cells can also reduce antigen escape.

CAR-T cell therapies targeting multiple antigens have improved antigen coverage, addressing the issue of antigen heterogeneity to some extent. Currently, multi-target CAR-T cells (Figure 3, relevant clinical data are shown in Table 1) are mainly achieved through the following approaches:

### 5.1. Tandem CAR-T (TanCAR-T)

TanCAR-T cells achieve multi-target recognition by combining two distinct antigen recognition domains into a single CAR molecule. A phase I clinical trial evaluated CAR-T cells targeting CD20 and CD19 in 22 patients with relapsed or refractory B-cell malignancies, achieving an overall response rate of 82% by day 28 [63]. No CD19 loss was observed in relapsed patients, suggesting that dual-specific CAR may reduce target antigen downregulation and improve clinical responses [63].

Although TanCAR-T compensates for some of the limitations of single-target CAR-T cells, the increased structural complexity of CAR requires simultaneous consideration of the spatial relationship between the two scFvs when recognizing their respective antigens, as well as balancing the affinities of the two recognition domains to ensure effective targeting of both antigens. Tong et al. reported that TanCAR-T cells targeting both CD19 and CD20 can simultaneously cover dual tumor targets, form stable immune synapse structures, and retain memory-associated markers [64]. In a phase I clinical trial targeting CD19 and CD22 for the treatment of B-cell lymphoma, it was observed that the CD22 scFv triggered less cytokine secretion compared to the CD19 scFv, suggesting potential differences in the efficacy of CAR-T cells against different targets [19]. Additionally, the length and flexibility of the linker between scFvs influence the cytotoxicity of tandem CAR-T cells. For CAR-T cells targeting GRP78 and CD123 simultaneously, shorter and more flexible linkers better maintained the spatial conformation of the dual CAR, enhancing their targeting ability and stability [79]. Therefore, future research on tandem CAR will need to further refine the structural design to achieve optimal targeting efficiency.

### 5.2. Dual CAR-T

Dual CAR-T cells co-express two separate CAR structures in a single T cell, delivering two different signaling pathways. One challenge is selecting the appropriate co-stimulatory domains and signaling regions for synergistic activation of two targets. In preclinical models for treating pediatric rhabdomyosarcoma with FGFR4 and CD276, and B-ALL with CD19/CD20, dual CARs with diverse co-stimulatory domains (CD28 and 4-1BB) showed better persistence and anti-tumor activity than dual CARs with the same co-stimulatory domains [71,80]. However, in a mouse model targeting GPRC5D/BCMA for multiple myeloma, dual CARs with both 4-1BB co-stimulatory domains showed stronger anti-tumor effects [81]. Hirabayashi et al. developed dual CAR-T cells targeting GD2 and B7-H3, which exhibited strong and durable anti-tumor activity in heterogeneous neuroblastoma [82]. They used different co-stimulatory signals (CD28 and 4-1BB) for each CAR while sharing the same CD3ζ chain, successfully combining the advantages of both co-stimulatory molecules and preventing T cell exhaustion after antigen stimulation [82].

### 5.3. Trivalent CAR-T Cell

In addition to dual-target CAR-T cells, researchers have also designed trivalent CAR-T cells. In the treatment of B-cell malignancies, CD19, CD20, and CD22 are commonly used as combinational target antigens. Fousek et al. reported that CAR-T cells targeting these three antigens simultaneously can effectively eliminate tumor cells that have relapsed and become CD19-negative after CD19 CAR-T treatment; these, CAR-T cells can still form effective immune synapses with tumor cells [83]. Schneider and colleagues developed trispecific CD19-CD20-CD22-targeted CAR-T cells that rapidly and efficiently rejected leukemia and lymphoma tumors in vivo, whereas single-target CAR-T cells failed to inhibit tumor progression [84].

### 5.4. Combining Different Single-Target CAR-T Therapies

This approach is a cocktail-style cell therapy in which CAR-T cell lines targeting two or more different antigens are either mixed or administered sequentially. In 2020, Pan et al. conducted a phase I trial involving 20 patients and found that sequential infusion of CD19 and CD22 CAR-T cells significantly improved long-term overall survival and disease-free survival in patients with post-transplant relapsed B-ALL [73]. Among the 20 patients, 17 (85%) achieved sustained remission, with a 1-year leukemia-free survival (LFS) rate of 79.5% and an overall survival rate of 92.3% [73]. These preliminary results confirmed the safety and feasibility of this sequential strategy. To further validate the long-term efficacy of sequential CD19-CD22 CAR-T therapy, a phase II study was conducted. Among 79 patients who received sequential infusions of CD19 and CD22 CAR-T cells, the 18-month event-free survival (EFS) rate was 79% (95% CI: 66-91%), the disease-free survival (DFS) rate was 80%, and the overall survival (OS) rate was 96% [74]. The treatment was well tolerated with manageable adverse events, confirming the safety of this sequential therapy. A recent clinical study evaluated the efficacy and safety of CD22 CAR-T therapy in patients with relapsed large B-cell lymphoma who had progressed after CD19 CAR-T treatment [20]. Compared with the median survival of only 6 months after CD19 CAR-T therapy failure, the 2-year survival rate of CD22 CAR-T therapy after sequential infusion at the maximum tolerated dose reached 52%, indicating that sequential treatment can benefit some patients who have failed single-target therapy [20].

Although current data suggest that dual-target therapy has the potential to address antigen heterogeneity, it is still necessary to consider the persistence of CAR-T cells under stimulation with multiple antigens and which multi-target strategy holds the greatest advantage. In addition, introducing multiple genes into CAR-T cells increases the vector size, reduces transduction efficiency, and may generate heterogeneous CAR-T subpopulations expressing only one scFv. This may lead to the selective expansion of a dominant subgroup during treatment [85]. James et al.’s leucine zipper-based screening system can isolate cells expressing two CARs, thereby effectively killing heterogeneous tumors [85]. In summary, more long-term follow-up data are needed to evaluate the effectiveness of multi-target CAR-T therapies.

## 6. Reprogramming the TME to Activate Endogenous Immunity

Although structural optimization of CAR and the design of multi-target strategies provide approaches to address tumor antigen heterogeneity, these methods are still insufficient to fully overcome this challenge [19,86]. The success of CD19-targeted CAR-T therapy can be partly attributed to the uniformly high expression of this target on the surface of tumors. Additionally, the non-specific toxicity to B cells caused by CD19-targeted CAR-T therapy can be managed by immunoglobulin supplementation [87]. However, for most tumor types, especially solid tumors, identifying a target that is both widely expressed and acceptable in terms of safety remains a significant challenge due to the extreme heterogeneity and ongoing dynamic changes in antigen expression [9]. The high diversity of T cell receptors (TCRs) enables CAR-T and endogenous T cells to effectively cope with the spatiotemporal variability of tumor antigens, which is crucial for eliminating antigen-escape variants [88]. In addition, researchers have found that non-cytotoxic T cells, such as Th9 cells and neutrophils, also play a key role in eliminating antigen-negative tumors [89,90].

**Figure 4:**
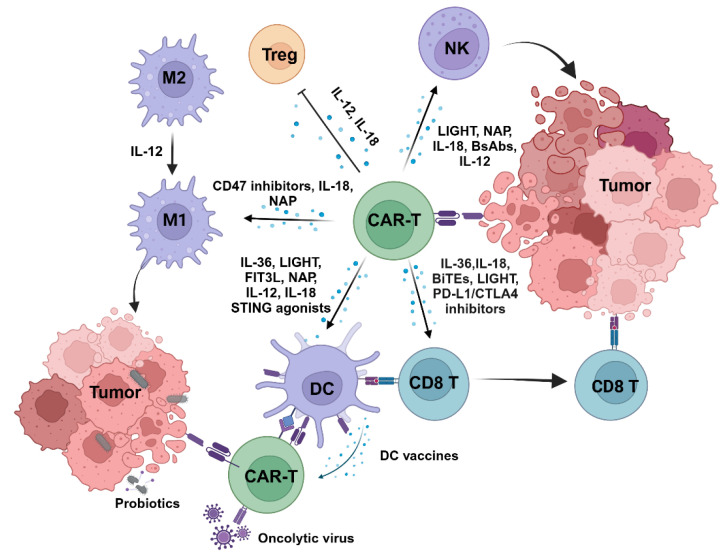
CAR-T cells secrete different immunoregulatory molecules or combine with vaccines, oncolytic viruses, or probiotics to reshape the TME and promote the cytotoxic activity of endogenous CD8 T cells and NK cells, thereby enhancing the overall immune response and eliminating antigen-negative tumors.

Based on this, more and more studies are focusing on the interactions between CAR-T cells and other cells in the TME and attempts to develop CAR-T cells’ ability to activate the endogenous immune system (Figure 4, relevant clinical data are shown in Table 2). Kaminski et al. found that in patients receiving Kymriah, a CAR-T therapy targeting CD19, endogenous CD8 T cells were activated in an IL-15 and IL-2-dependent manner, and expressed activation markers for both T cells and natural killer (NK) cells [91]. Ramos et al. analyzed immune responses to known tumor-associated antigens in the peripheral blood of patients before and after CD19 CAR-T cell infusion, and they observed no differences in the frequency of precursor T cells responding to these antigens before and after infusion [92]. Some reports have even suggested that CAR-T therapy may trigger a compensatory immunosuppressive response [25]. Therefore, whether second-generation CAR-T therapies based on dual-target CAR structures can trigger therapeutically meaningful activation of endogenous immunity remains to be investigated. Third-generation CAR-T cells add an additional costimulatory domain (such as CD28, 4-1BB, or OX40) to the second-generation CAR-T, enhancing T cell activity and persistence. Fourth-generation CAR-T cells (also known as “TRUCKs” or “armored CAR-T cells”) are typically designed with additional functions, such as secreting specific cytokines or immunomodulatory molecules, to actively remodel the TME and fully activate the endogenous immune system [50], which brings new possibilities for overcoming antigen heterogeneity.

### 6.1. Cytokine-Armed CAR-T Cells

One effective strategy for modifying the TME has been to target the cytokine network. CAR-T cells armed with cytokines have demonstrated benefits in preserving stemness, reprogramming T cell metabolism, reducing exhaustion phenotypes, improving immune cell trafficking, and regulating the immune microenvironment [93,94]. CAR-T cells secreting IL-36 activate the Myd88-dependent pathway through autocrine mechanisms, enhancing their own proliferation and persistence while producing more IFN-γ and TNF-α, which support the activity of endogenous T cells and DCs [95]. IL-18 also has a positive role in remodeling the TME and exhibits good synergy when used in combination with CAR-T [96,97]. Recent studies have found that compared to conventional CAR-T cells, CAR-T cells secreting IL-18 can better eliminate low-antigen density myeloma cells by reprogramming the immune microenvironment [98]. By expressing tumor necrosis factor superfamily member 14 (LIGHT), CAR-T cells targeting prostate-specific membrane antigen (PSMA) can induce a variety of LTβR-related chemokines and adhesion molecules, remodeling the vasculature and lymphatic structures within the tumor, thereby enhancing the infiltration of DCs, NK cells, and T cells [99]. IL-12 possesses broad pro-inflammatory and antitumor effects. It promotes T cell differentiation toward a Th1-type immune response, inhibits Treg function, enhances antigen presentation, and reprograms tumor-associated macrophages (TAMs) to an M1 phenotype [100,101]. Based on these properties, IL-12 is often used in combination with CAR-T cells, playing a powerful role in the treatment of both solid tumors and hematologic malignancies [102,103].

Although cytokines and CAR-T cells have strong synergistic effects, the therapeutic window of systemically administered cytokines in combination therapy is narrow and there is a risk of severe cytotoxicity. In addition, intratumoral injection is not suitable for microlesions and deep tumors. Finding ways to confine their effects controllably within the tumor remains an area of ongoing exploration. For example, IL-12 can be conjugated with a domain that binds to tumor stromal collagen, leveraging the abundant collagen in the stroma of solid tumors, thereby achieving targeted intratumoral delivery [103]. Nanotechnology can also be used to attach IL-12 to the surface of CAR-T cells, allowing for effective release in response to tumor antigen stimulation [104]. Andreas and his colleagues designed a CAR by inserting IL-12 into the extracellular portion of a CD28-ζ CAR. This modification endowed CAR-T cells with an NK cell-like phenotype, exhibiting high expression of CD94, CD56, and CD62L [105]. Unlike T cells, NK cells’ cytotoxic activity does not rely on MHC molecules or specific antigens [106,107]. Instead, it is dynamically regulated by the balance between activating and inhibitory receptors. Compared to conventional CAR-T cells, IL-12-CAR-T cells displayed antigen-independent and NK cell-like cytotoxic activity, thereby being able to eliminate antigen-negative cancer cells [105]. Due to NK cells’ diverse killing mechanisms and antigen-independent nature, they can respond more flexibly to antigen heterogeneity than T cells [108]. Engineered CAR-NK cells (expressing CAR molecules on NK cells) have shown potent antitumor activity and safety [109,110]. Ma and his team inserted the IL-15 gene into the 3′ UTR of the CAR-T cell’s IFN-γ gene, enabling IL-15 expression to be controlled by the IFN-γ promoter [111]. This allows IL-15 to be released only when CAR-T cells are activated, demonstrating effective tumor clearance in low-antigen density models. To prevent CAR-T cell overactivation and cytokine release syndrome (CRS), researchers have introduced an inducible suicide switch based on caspase-9 (inducible caspase 9, iC9) into CAR-T cells. This system can be activated by the chemical inducer AP1903, which induces iC9 dimerization and leads to CAR-T cell apoptosis [112]. This approach has demonstrated good safety in both preclinical animal models and clinical trials, can rapidly clear circulating CAR-T cells [113,114,115], and has been shown to effectively reduce the incidence of CRS when combined with IL-15 [116]. In addition, CAR-T cells can be engineered to express receptors like EGFR that are detectable by monoclonal antibodies. When used in combination with monoclonal antibodies, these receptors enable the effective clearance of overactivated CAR-T cells through antibody-dependent cellular cytotoxicity (ADCC) or complement-dependent cytotoxicity (CDC) [117].

### 6.2. ICI-Armed CAR-T Cells

Inhibitory molecules expressed by tumor cells (such as PD-L1 and CD86) bind to checkpoint receptors on immune cells, thereby restricting anti-tumor immune responses. Consequently, immune checkpoint inhibitors (ICI) have been widely used to reinvigorate immune responses [118]. However, since immune checkpoints also play a role in maintaining immune homeostasis, systemic administration can trigger uncontrollable inflammatory reactions. To address this issue, CAR-T cells have been engineered to secrete immune checkpoint inhibitors. Chen et al. constructed a CAR-T cell with autocrine scFv targeting PD-1 and TREM2, which limits the function of MDSCs and TAMs. In a mouse colorectal cancer model, this CAR-T cell exhibited strong anti-tumor activity [119].

CD47 is known as the “don’t eat me” signal, which interacts with the SIRPα receptor on phagocytes (such as macrophages) to help cells evade phagocytosis [120]. Tumor cells often overexpress CD47 to escape immune system attacks, making CD47 an important target in cancer therapy [120]. Delivering CD47 inhibitors (SIRPα-Fc, CV1) using CAR-T cells activates the innate immune system within the TME, reprogramming macrophages from an M2 to an M1 phenotype and enhancing the ADCC function of myeloid cells in vitro and in vivo, thereby reversing the immunosuppressive effects of monocytic cells [121,122]. However, the use of CD47 antibodies may also lead to macrophage-mediated phagocytosis of T cells since T cells also express CD47. To address this, Yamada-Hunter’s team designed a CD47 variant, when CAR-T cells express this variant, they are not phagocytosed by macrophages even in the presence of CD47 antibodies, making the combination of CD47 antibodies and CAR-T cells more efficient [123].

In addition to exploring different secreted molecules targeting immune checkpoints, studies have also focused on the nature of the inhibitors. Besides secreting monoclonal antibodies and scFvs, single-domain antibody fragments (VHH) or VHH fusion proteins are also used. Due to their small protein size and good stability, these molecules are more easily secreted in a functional form. CAR-T cells expressing anti-PD-L1 or anti-CTLA-4 nanobodies have shown improved persistence and anti-tumor activity [124] (relevant clinical data are shown in Table 2).

### 6.3. CAR-T Cells Armed with Other Immunostimulatory Molecules

Combining highly immunogenic substances with CAR-T cells can better engage the innate immune system and promote epitope spreading. Delivery of CAR-T cells along with the STING agonist enhances endogenous immune responses against non-CAR-targeted antigens, driving BATF3 DC1-dependent tumor control, creating a pro-inflammatory microenvironment and epitope spreading [125,126,127]. Co-administration with a STING agonist improves the chemokine profile of the tumor microenvironment and promotes CAR-T cell homing [128]. Pattern recognition receptors (PRRs) are crucial molecules in the immune system expressed on innate immune cells such as macrophages, DCs, and neutrophils [129]. PRRs recognize pathogen-associated molecular patterns (PAMPs) and damage-associated molecular patterns (DAMPs), subsequently activating the NF-κB signaling pathway, leading to metabolic reprogramming of immune cells, secretion of pro-inflammatory cytokines, and initiation of adaptive immunity [129]. Wang and his team engineered CAR-T cells to secrete a non-coding RNA RN7SL1 through EVs. After myeloid cells and DCs take up EVs, endogenous immune responses are activated through the RIG-1 and MDA5 pathways [130]. This method alleviated immunosuppression and inhibited tumor progression. Helicobacter pylori neutrophil-activating protein (NAP) can trigger innate immune responses, induce DC maturation, and promote Th1 cell polarization, creating a pro-inflammatory environment [131]. Jin et al. developed CAR-T cells that express NAP (CAR (NAP) T cells). The release of NAP induced a broader pro-inflammatory effect, attracting neutrophils, M1 macrophages, NK cells, and DCs to the tumor site [131]. This robust, multi-faceted cytotoxic response induced bystander effects and promoted epitope spreading in tumor models.

Bispecific T cell engagers (BiTEs) are a subclass of bispecific antibodies specifically designed to guide T cells to tumor cells. BiTEs are typically small molecular proteins composed of two scFvs. One scFv specifically binds to the CD3 molecule on the surface of T cells, while the other scFv binds to a specific antigen on the surface of tumor cells. This dual binding brings T cells and tumor cells into close physical proximity, promoting T cells to recognize and kill tumor cells [132]. Engineering CAR-T cells to secrete BiTEs not only enhances their own cytotoxicity but also redirects endogenous T cells, promoting T cell homing and generating long-term immunity [133,134]. Similarly, Pascual-Pasto and colleagues engineered GPC2-targeting CAR-T cells to secrete bispecific innate immune cell engagers (BiCE) that simultaneously target the GD2 and CD16a receptors, thereby activating bystander NK cells and macrophages in the tumor microenvironment [135].

CAR-T cells can also serve as “mini-pharmacies” carrying anti-tumor drugs. In the synthetic enzyme-armed killer (SEAKER) cell therapy platform developed by Gardner and his colleagues, CAR-T cells are designed to secrete prodrug-activating enzymes in an antigen-dependent manner, working in synergy with systemically administered prodrugs [136]. As CAR-T cells proliferate, the enzyme concentration increases locally in the tumor and activates the prodrugs, which can kill antigen-negative cancer cells without being affected by the immunosuppressive tumor microenvironment or T cell exhaustion [136]. This approach offers a new concept for CAR-T cell development.

### 6.4. Targeting Dendritic Cells or Combining CAR-T Therapy with Vaccines

Dendritic cells (DCs) provide activation signals for CD8 T cell activation through antigen cross-presentation pathways, thereby initiating adaptive immunity. However, various immunosuppressive factors within the TME interfere with the function of DCs, hinder immune surveillance, and promote tumor progression [137]. It is reported that a strong antigen-presenting ability of type I DCs is associated with improved efficacy of CAR-T therapy [138]. CD40 is a type II transmembrane protein expressed on DCs, and CD40 signaling increases the expression of MHC molecules and costimulatory molecules on DCs, thereby enhancing their antigen presentation function [139]. CAR-T cells with constitutive expression of CD40L enhance DC activity through the CD40/CD40L pathway and recruit tumor-specific endogenous T cells [140]. Lai et al. constructed HER2-targeted CAR-T cells that secreted FMS-related tyrosine kinase 3 ligand (Flt3L), a growth factor that promotes DC development, in combination with the immune adjuvant poly (I:C) and the 4-1BB agonist to activate cDC1s and induce type I IFN pro-inflammatory characteristics [141]. Compared to traditional CAR-T cells, Flt3L-secreting CAR-T cells successfully induced antigen epitope spreading beyond the antigen recognized by CAR-T cells [141].

Although the invention of CAR allows T cells to bypass MHC restrictions, it also results in the absence of sufficient activation signals, particularly the co-stimulation provided by DC to T cells, which is essential for effective T cell function. As mentioned earlier, oncolytic viruses can promote CAR-T cell expansion by stimulating virus-specific TCRs. In addition to sensitizing TCRs, Professor Darrell Irvine’s team developed an amphiphilic ligand that expresses the antigen recognized by CAR and can be inserted into the cell membrane surface of DCs [142]. This strategy enables DCs to present antigen peptides to CAR-T cells in the lymph nodes, leading to significant in vivo proliferation of CAR-T cells and enhancing their anti-tumor efficacy [142]. This process mimics the natural antigen presentation process, reprogramming the function of CAR-T cells. Compared to the TME, the lymph node microenvironment is conducive to T cell activation and proliferation, providing the three essential signals required for full T cell activation—something that cannot be achieved solely by targeting tumor cells.

DC vaccines can enhance the proliferation and activation of CAR-T cells [143]. Their combination can also reshape TME and promote epitope spreading [144]. In Ma’s study, CAR-T cells activated by DC vaccines underwent metabolic reprogramming, secreting large amounts of IFN-γ, which formed a positive feedback loop with IL-12 secreted by DCs [144]. This enhanced the antitumor potential of CD8 T cells and promoted the differentiation of tumor-infiltrating lymphocytes (TILs) into the Th1 subtype, effectively eliminating heterogeneous tumors.

In addition to the aforementioned amphiphilic ligand, using nanoparticle-encapsulated RNA vaccines can also be phagocytosed by DCs, which then express the CAR target antigen on their surface to stimulate CAR-T cell proliferation in vivo [145] (relevant clinical data are shown in Table 3). A clinical trial demonstrated that the combination strategy of CAR-T cell therapy targeting CLDN6 with amplifying RNA vaccine showed good safety [146].

### 6.5. Combining CAR-T Therapy with Oncolytic Virus

Oncolytic viruses are a class of viruses that selectively kill tumor cells. They leverage the deficiencies in tumor cells’ antiviral defense mechanisms, specific receptors, and rapid growth characteristics to preferentially lyse cancer cells. During this process, they stimulate surrounding immune cells to produce various inflammatory cytokines and chemokines and induce immunogenic cell death of the target cells [147]. Due to these characteristics, oncolytic viruses are excellent partners for combination with immunotherapy. Engineered oncolytic viruses have been designed to carry various effector molecules, such as secreting hyaluronidase to dissolve the extracellular matrix and promote CAR-T cell infiltration, carrying specific antigens to increase target antigen abundance after infecting tumor cells, or delivering IL-15 to modulate the tumor immune microenvironment and help CAR-T cells overcome the immunosuppressive microenvironment [148,149,150].

However, the inflammatory environment generated by oncolytic viruses does not always favor CAR-T cell function, as it depends on the specific nature and context of the inflammation. Evgin et al. injected an oncolytic virus expressing IFNβ into B16EGFRvIII tumors in an immunocompetent mouse model, followed by administration of EGFRvIII-targeting CAR-T cells. Although the oncolytic virus altered the chemokine profile in TME, the β-interferon (IFNβ) produced in vivo led to apoptosis of the IFN-sensitive CAR-T cells [151]. Based on this observation, the team explored a new approach to combining CAR-T cells with oncolytic viruses. They found that injecting an oncolytic virus carrying mIFNβ after CAR-T cell transfer effectively increased the levels of CD8 CAR-T cells and enhanced antitumor efficacy without exhausting CAR-T cells [152]. This finding suggests that oncolytic viruses may be employed as vaccines to stimulate virus-specific TCRs on CAR-T cells, hence activating CAR-T cells.

In addition to the above synergistic mechanisms, McFadden and colleagues discovered that when CAR-T cells are used to deliver myxoma virus (MYXV), the IFNγ-protein kinase B (AKT) signaling pathway generated by T cells works in concert with the MYXV-induced M-T5-SKP-1-VPS34 signaling pathway to cause autophagy in tumor cells [153]. During MYXV-mediated autophagy, cancer cells near the therapeutic target are destroyed through a “bystander killing” effect, regardless of antigen expression [153]. This finding offers new possibilities for addressing tumor antigen heterogeneity.

### 6.6. Combining CAR-T Therapy with Probiotics

Using the natural tumor-targeting tendencies of certain bacteria, Vincent’s team engineered Escherichia coli Nissle 1917 to carry a tag, which is then released at the tumor site [154]. The tag’s one end carries GFP, which CAR-T cells can recognize, while the other end targets surface and matrix proteins in the TME [154]. This allows the redirection of endogenous T cells while guiding CAR-T cells, achieving a targeting effect independent of tumor-associated antigens.

## 7. Conclusions

A prerequisite for the successful cytotoxicity of CAR-T treatment is the effective recognition of tumor antigens by CAR molecules. The spatiotemporal heterogeneity of tumor antigens has always been a significant barrier in CAR-T therapy. Optimizing the structure of CAR to incorporate more regulatory modules enables more orderly and balanced signal transduction. Fourth-generation CAR-T and combination therapies activate multiple branches of the immune system to compensate for the shortcomings of single cytotoxic effects. As the role of CAR-T shifts from killer to regulator, this also prompts reconsideration and re-evaluation of pre-treatment lymphodepletion in CAR-T therapy, as well as how to guide, regulate, and confine this immunomodulatory effect. This requires a deeper understanding of the interactions and crosstalk between CAR-T cells and the cells in the TME. Future efforts will need to focus on a more thorough analysis and evaluation of the safety and efficacy of these approaches in humans.

## Figures and Tables

**Table 1:** Clinical study of dual-target CAR-T.

Target Antigen	Disease	Method	Clinical Stage	Result (*n* = Treated Patients)	Grade 3–4 CRS	Grade 3–4 ICANS	Clinical Trial Registration Number	Ref.
CD20/CD19	B-CLL/B-NHL	Tandem CAR-T	phase I	ORR = 82% CRR = 64% (*n* = 22)	5%	14%	NCT03019055	Shah et al. [63]
CD20/CD19	R/R NHL	Tandem CAR-T	phase I/II	ORR = 79% CRR = 41% 12-month PFS = 64% (*n* = 28)	14%	none	NCT03097770	Tong et al. [64]
CD20/CD19	R/R NHL	Tandem CAR-T	phase I/II	ORR = 90% CRR = 70% (*n* = 10)	9%	18%	NCT04723914	Wang et al. [65]
CD20/CD19	R/R NHL	Tandem CAR-T	phase I	ORR = 90% CRR = 70% (*n* = 10)	None	None	NCT04007029	Larson et al. [66]
CD20/CD19	R/R NHL	Tandem CAR-T	phase I/II	ORR = 78% PFS = 27.6 months (*n* = 58)	10%	2%	NCT03097770	Zhang et al. [67]
CD22/CD19	R/R B-ALL	Sequential CAR-T Tandem CAR-T	phase I	Sequential CAR-T: CRR = 95.2% (*n* = 21) Tandem CAR-T: CRR = 98% (*n* = 51)	Sequential CAR-T:19% Tandem CAR-T:13.7%	Tandem CAR-T:2%	NCT03614858	Liu et al. [68]
CD22/CD19	B-ALL	Dual CAR-T	phase I	CRR = 83% (*n* = 12)	None	8%	NCT02443831	Ghorashian et al. [69]
CD22/CD19	r/r B-ALL	Tandem CAR-T	phase I	CRR = 100% (*n* = 47)	17%	2%	NCT03614858	Cui et al. [70]
CD22/CD19	B-ALL	Dual CAR-T	phase I	CRR = 60% (*n* = 20)	15%	5%	NCT03448393	Shalabi et al. [71]
CD22/CD19	R/R B-ALL	Tandem CAR-T	phase I	CRR = 89% (*n* = 9)	None	None	NCT03233854	Srinagesh et al. [72]
CD22/CD19	R/R B-ALL	Sequential CAR-T	phase I	CRR = 85% (*n* = 20)	None	5%	ChiCTR-OIB-17013670	Pan et al. [73]
CD22/CD19	R/R B-ALL	Sequential CAR-T	phase II	18m EFS = 79% DoR = 80% DFS = 80% OS = 96% (*n* = 79)	19%	5%	NCT04340154	Pan et al. [74]
CD22/CD19	LBCL	Sequential CAR-T	phase I	ORR = 68% CRR = 63% (*n* = 40)	None	None	NCT04088890	Frank et al. [20]
BCMA/CD19	R/R MM	Tandem CAR-T	phase II	ORR = 95% sCR = 43% CRR = 14% (*n* = 21)	9%	None	ChiCTR-OIC-17011272	Yan et al. [75]
BCMA/CD19	R/R MM	Tandem CAR-T	phase I	sCR =86 (*n* = 22)	None	None	unmentioned	Qiang et al. [76]
BCMA/CD19	R/R MM	Combined infusion	phase II	ORR = 92% CRR = 60% (*n* = 62)	10%	3%	ChiCTR-OIC-17011272	Wang et al. [77]
BCMA/CD19	R/R MM	Tandem CAR-T	phase I/II	ORR = 92% PFS = 19.7 months (*n* = 50)	8%	None	ChiCTR2000033567	Shi et al. [19]
BCMA/GPRC5D	R/R MM	Tandem CAR-T	phase I	ORR= 86% CRR = 62% (*n* = 21)	None	5%	NCT05509530	Zhou et al. [78]

Abbreviations: ORR, overall response rate; CRR, complete remission rate; sCR, stringent complete remission; PFS, progression-free survival; DoR, duration of remission; DFS, disease-free survival; OS, overall survival; CRS, cytokine release syndrome; ICANS, immune effector cell-associated neurotoxicity syndrome; PFS, progression-free survival.

**Table 2:** Clinical trials of CAR-T therapy related to remodeling the TME.

Target Antigen	Disease	Method	Status	Clinical Trial Registration Number
MUC16	Ovarian cancer, primary peritoneal cancer, fallopian tube cancer	IL-12	Unknown	NCT02498912
EGFR	Metastatic colorectal cancer	IL-12	Unknown	NCT03542799
Nectin4/FAP	Breast cancer, colorectal cancer, non-small cell lung cancer, pancreatic ductal adenocarcinoma	IL7 and/or CCL19 or IL12	Unknown	NCT03932565
CD19	Refractory or relapsed CD19-positive leukemia or lymphoma	IL-12	Recruiting	NCT05343376
CD19	ALL	IL-18	Recruiting	NCT05899024
CD371	AML	IL-18	Recruiting	NCT06012758
CD19	R/R B-NHL B-CLL	IL-18	CR = 55%	NCT04684563
GPC3	GPC3-positive solid tumor	IL-15	Recruiting	NCT04377932 NCT05103631
GD2	R/R NB or R/R OS	IL-15	Recruiting	NCT03721068
MUC1	MUC1 Positive Advanced Malignancies	PD-1/CTLA4 antibody	Unknown	NCT03179007
EGFR	EGFR Positive Advanced Malignancies	PD-1/CTLA4 antibody	Unknown	NCT03182816
MSLN	MSLN Positive Advanced Malignancies	PD-1 antibody	Unknown	NCT03615313
MSLN	MSLN Positive Advanced Malignancies	PD1/CTLA-4 nanobody	Recruiting	NCT06248697
MSLN	MSLN Positive Advanced Malignancies	PD-1 nanobody	Recruiting	NCT04489862

Abbreviations: MUC16, mucin16; R/R NB, relapsed/refractory neuroblastoma; R/R OS, relapsed/refractory osteosarcoma.

**Table 3:** Clinical trials of CAR-T therapy combined with vaccines.

Target Antigen	Disease	CAR-T	Vaccine	Results	NCT Number
Claudin6	GCT, EOC, CUP, DSRCT	Claudin6-CAR T	RNA-LPX	Well tolerated	NCT04503278 [145]
CD19	B-NHL	CMV-Specific CD19-CAR T	CMV-MVA Triplex	Recruiting	NCT05801913
GD2	Sarcoma and Neuroblastoma	iC9-GD2-CAR-VZV-T	Varicella-zoster vaccine	Recruiting	NCT01953900
CD19	B-NHL	CMV-Specific CD19-CAR T	CMV-MVA triplex vaccine	Recruiting	NCT05432635
GD2	Relapsed/Refractory Melanoma	tvs-CTL	normal vaccine	completed	NCT02482532

Abbreviations: RNA-LPX, ribonucleic acid lipoplexes; CMV, cytomegalovirus; VZV, varicella-zoster virus; MVA, modified vaccinia Ankara; tvs-CTL, activated T cells enriched for vaccine-specific cytotoxic T-lymphocytes.

## Data Availability

No new data were created or analyzed in this study.
